# Insights into the Effects of Carbamylated Erythropoietin on Schwann Cells in Peripheral Nerve Injury

**DOI:** 10.3390/ijms27104434

**Published:** 2026-05-15

**Authors:** Zhenzhen Wu, Ting Chak Lam, Shanthini Kalimuthu, Yiu Yan Leung

**Affiliations:** Oral and Maxillofacial Surgery, Faculty of Dentistry, The University of Hong Kong, Hong Kong, China; cecewu@hku.hk (Z.W.); u3507413@connect.hku.hk (T.C.L.); shan97@connect.hku.hk (S.K.)

**Keywords:** peripheral nerve injuries, CEPO, NGF expression, nerve repair and regeneration

## Abstract

Recent advancements in biology and medicine have facilitated the progress of nerve regeneration that markedly improves the treatment of peripheral nerve injuries, enhancing outcomes and recovery rates. It has been reported that erythropoietin (EPO) is currently being studied as a potential agent for neural repair. However, much evidence has confirmed that EPO treatment can induce systemic adverse effects in the clinical fields, including coronary stent thrombosis and deep vein thrombosis. Herein, a derivative of EPO without any hematopoietic activities, which is named carbamylated erythropoietin (CEPO), has been synthesized and investigated for its effects on peripheral neural repair both in vitro and in vivo. The in vitro experimental results demonstrated that CEPO enhanced Schwann cell viability, proliferation, migration, and nerve growth factor (NGF) expression, while the optimal concentration of CEPO was found to be 25 μg/mL. The in vivo observations at 21 days post-injection indicated that the CEPO group exhibited a significant functional improvement in the sciatic nerve injury model, guiding regrowing axons across the injury site. Thus, CEPO serves as a promising candidate or adjunctive strategy for peripheral nerve injuries, demonstrating promising clinical applications and potential for enhancing Schwann cell viability, proliferation, and migration, as well as anticipated nerve axon development.

## 1. Introduction

Nerve injury involving the lingual nerve (LN) and inferior alveolar nerve (IAN) is a common complication of routine oral surgical procedures, such as the removal of wisdom teeth and enucleation of odontogenic cysts. According to Seddon’s classification, peripheral nerve injuries are categorized as neuropraxia, axonotmesis, and neurotmesis, with ascending severity of injury [1,2]. The variation in types of nerve damage plays an important role in influencing the innate healing response of repair or regeneration, and consequently affects the choice of surgical methods for the damaged nerve. Patients who suffer from permanent nerve injury report various symptoms, including mild hypoesthesia, paresthesia, and chronic pain [3,4,5]. Persistent deficits of the LN and IAN have been shown to correlate negatively with the oral health-related quality of life (OHRQoL) [6,7,8]. Furthermore, various studies have demonstrated that patients with trigeminal neurosensory deficits following third molar surgery experience significantly reduced life satisfaction and have an increased prevalence of depressive symptoms, while these conditions are even worse in the elderly [9,10,11].

After a traumatic peripheral nerve injury, Schwann cells play an essential role in contributing to the intrinsic healing capacity [12,13,14]. They orchestrate the consequential healing events immediately after nerve injury, irrespective of whether the injury is axonotmesis or neurotmesis, from mediating Wallerian degeneration to optimize the cellular environment for healing, to the formation of functional regenerative guidance tracks for axonal growth [15,16]. Axonal regeneration is impeded by the formation of glial scars and the obliteration of the endoneurium from subsequent fibrosis, or, in more severe cases, by the formation of neuromas, posing further obstacles to successful nerve healing and regeneration. Failure in axonal interconnection between the proximal and distal stumps can result in misdirected reinnervation. Therefore, the outcome of functional recovery after neurotmesis with or without repair is generally poor [17,18].

Surgical intervention may be required to facilitate successful nerve regeneration and achieve functional recovery of the nerve [19,20]. Despite advances in microsurgical techniques and biomaterials, the repair of injured peripheral nerves remains a significant clinical challenge to surgeons [21,22,23]. Surgical modalities such as autologous nerve or vein grafting are widely regarded as the contemporary gold standard for the treatment of discontinuous defects in peripheral nerve injury. Nevertheless, it compromises the neural function of the donor sites post-operatively and has limitations regarding tissue availability [24]. Other surgical modalities, such as external neurolysis and direct suturing of nerves, are also highly technique-sensitive and demand substantial surgical expertise [25,26]. These shortcomings have driven researchers to investigate pharmaceutical alternatives to reduce the complexity of management and lower the threshold in treatment provision.

Erythropoietin (EPO), an approved drug by the Food and Drug Administration in 1989 for patients with chronic kidney disease with anemia and for anemic conditions secondary to cancer patients on chemotherapy and Zidovudine-treated HIV patients, has emerged as a promising candidate for neural repair [27,28]. Previous studies have demonstrated neuroprotective effects of EPO in both in vivo and in vitro models [29,30,31]. However, most investigations of its neuroprotective property have focused on the central nervous system, including applications in stroke and Alzheimer’s disease, whereas data on the impact of EPO on the peripheral nervous system remain scarce. Moreover, systemic adverse events related to EPO administration, such as coronary stent thrombosis and deep vein thrombosis, have been reported [32,33,34]. Under these circumstances, the clinical use of EPO has remained largely limited due to its hematological therapeutic indications rather than neurological applications.

Derivatives of EPO, such as carbamylated erythropoietin (CEPO), have been developed to retain its neuroprotective properties while eliminating hematological effects. Emerging evidence indicates that CEPO, a homolog of EPO, has similar neuroprotective effects to EPO without exerting any hematopoietic activities. Leist et al. [35,36] have confirmed the neuroprotective effects of CEPO in various animal models of neurotoxicity, such as ischemic stroke, sciatic nerve compression, spinal cord compression, and peripheral diabetic neuropathy [35,37]. Furthermore, many investigations have confirmed that CEPO has little or negligible erythropoietic activity in comparison to EPO, even after long-term administration of high doses, which makes CEPO a promising area for research and development in treating peripheral nerve injuries in patients with kidney disease [38,39,40]. However, its application to traumatic nerve injuries has not been proven. In addition, the production of CEPO is also not standardized and not commercially available, which presents a significant obstacle to further investigation of its neuroprotective potential.

**Figure 1:**
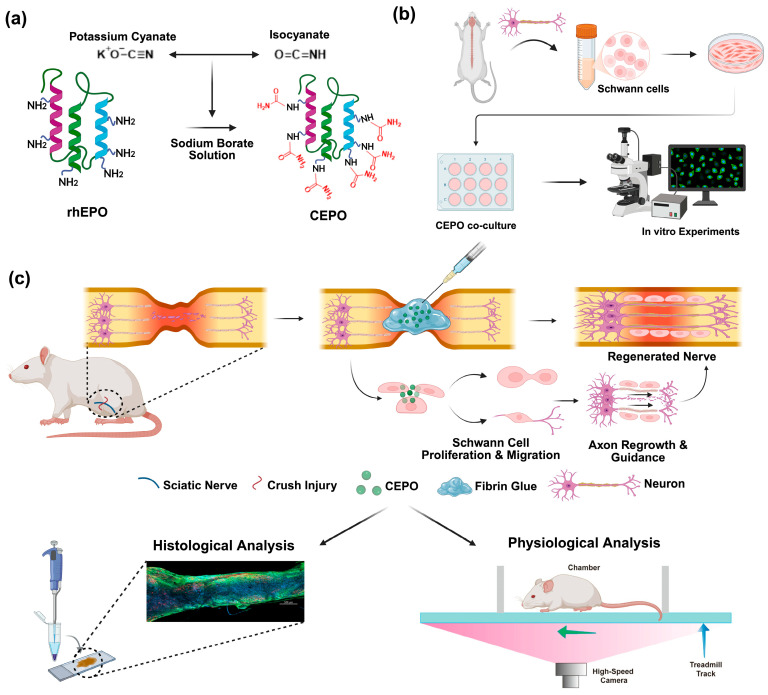
Schematic illustration of the experimental setup for CEPO and its in vitro and in vivo tests in the assessment of peripheral nerve regeneration. (**a**) Synthesis of CEPO from rhEPO by potassium cyanate in sodium borate solution. (**b**) Diagrammatic representation of experiments to determine the effect of CEPO on the Schwann cells in vitro. (**c**) The in vivo physiological and histological analysis in the sciatic nerve defect rat model with CEPO-fibrin glue injection upon crush injury.

By analyzing the effects of CEPO on Schwann cells and their function in nerve regeneration, this study assesses CEPO as a neuroprotective agent and investigates its therapeutic potential (Figure 1). Understanding how CEPO affects the behavior of Schwann cells is crucial to evaluating its therapeutic potential, since these cells are necessary for peripheral nerve repair [41,42,43,44]. Quantitative assays are employed to determine the effects of CEPO on Schwann cell migration and proliferation. Additionally, changes in nerve growth factor (NGF) levels, which are essential for neuronal survival, differentiation, and regeneration, are also evaluated using enzyme-linked immunosorbent assays (ELISA). The neuroprotective efficacy of CEPO is further assessed in vivo using a rat model of sciatic nerve crush injury, with the sciatic functional index (SFI) serving as a measure of functional recovery. Histological evidence of nerve repair, together with improved SFI scores, supports the therapeutic potential of CEPO. Collectively, these findings suggest that CEPO is a promising candidate for the treatment of neurodegenerative conditions and peripheral nerve injuries.

## 2. Results

### 2.1. Identification of Recombinant Human Erythropoietin (rhEPO) Transformation to CEPO

Carbamylation of EPO was performed as previously reported [35]. The successful synthesis of CEPO was verified using an enzymatic lysine digestion method, based on the principle that successful carbamylation of EPO leads to the conversion of lysine residues to homocitrulline. Endoproteinase Lys-C enzyme treatment was applied to both EPO and CEPO, as it can break the peptide linkages of lysine in EPO, resulting in two cleaved bands when verified using PAGE electrophoresis. However, due to the absence of free lysine residues, CEPO is not susceptible to Lys-C digestion and therefore produces a single distinct band with no detectable cleavage products.

**Figure 2:**
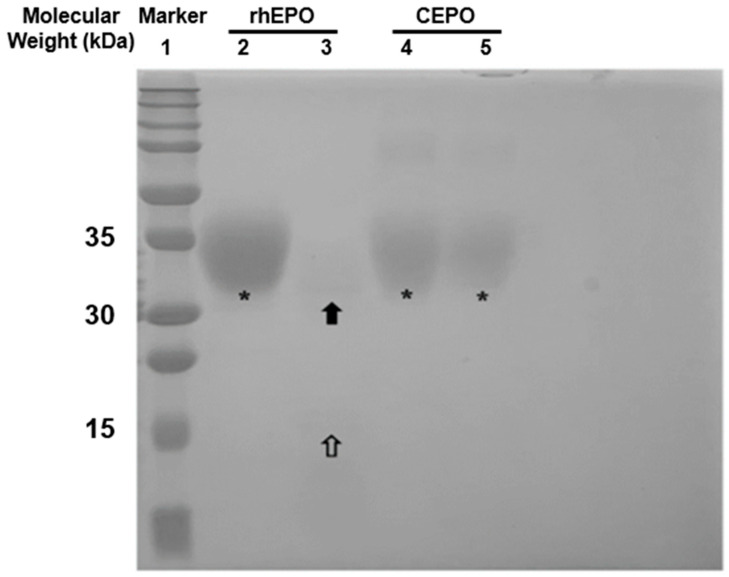
CEPO identification using SDS-PAGE electrophoresis. Lane 1: Marker ladder. Lane 2: Native EPO, a broad band of about 34 kDa (asterisk). Lane 3: Native EPO + endoproteinase Lys-C. approximately 31 kDa (black arrow) and 16 kDa (white arrow) fragments. Native EPO (Lane 2) and its Lys-C digest (Lane 3) act as internal controls demonstrating the expected cleavage. Lane 4: CEPO, a broad band of about 34 kDa (asterisk). Lane 5: CEPO + Endoproteinase Lys-C.

As shown in Figure 2, rhEPO and CEPO were processed in parallel on the same gel and served as internal controls for each other. The molecular weight marker in lane 1 provided reference bands at defined kDa values (including 31 and 34 kDa), which were used to estimate the apparent molecular weights of the intact and cleaved products. The second lane representing EPO and the fourth lane representing CEPO both displayed a broad band extending from a molecular weight of 31 to 34 kDa, as verified using the ladder markers in lane 1. The third and fifth lanes contained reaction products with endoproteinase Lys-C. In the third lane, EPO reacted with endoproteinase Lys-C, cleaving the peptide linkages and yielding two bands with molecular masses of approximately 31 kDa and 16 kDa. In the fifth lane, endoproteinase Lys-C was unable to cleave CEPO, thus still showing the broad band extending from around 31 to 34 kDa, similar to lane 4. No products around 16 kDa were observed in the fifth lane, as in the third lane, indicating successful carbamylation at lysine sites at the level of molecular weight and digestion pattern. While SDS-PAGE with Lys-C digestion does not provide complete structural elucidation, it confirms successful carbamylation of EPO as evidenced by the loss of Lys-C cleavage and preservation of a single band at 31–34 kDa.

### 2.2. In Vitro Biological Properties of CEPO

#### 2.2.1. Characterization of Schwann Cells

The cell lines of Schwann cells (SCs, ECACC, SCL4.1/F7) were characterized using immunocytochemical staining, as shown in Figure S1. Immunopositive staining with the S100β marker of the images illustrated the identity of Schwann cells. The Schwann-cell culture medium used in this study allowed solid attachment to the culture dish with significant growth and proliferation.

#### 2.2.2. Schwann Cells Cytotoxicity upon CEPO Treatments

**Figure 3:**
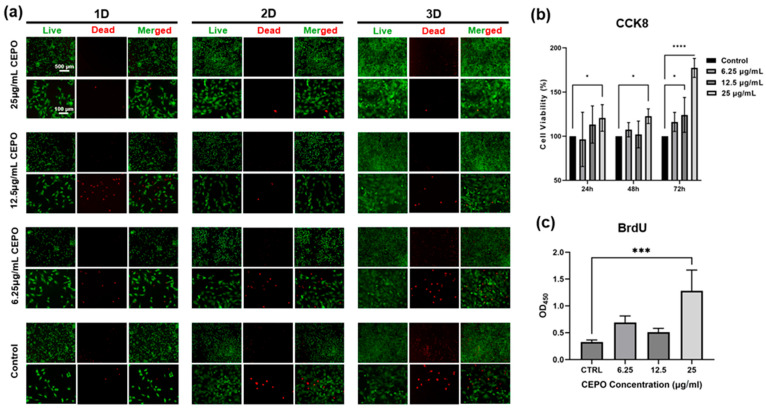
Schwann cell viability and proliferation under different concentrations of CEPO treatment. (**a**) Live/dead staining of the Schwann cells cultured with CEPO for 24 h, 48 h, and 72 h. Live cells were stained in green and dead cells in red. (**b**) The CCK-8 assay was used to demonstrate Schwann cell viability after seeding for 1, 2, and 3 days (*n* = 6). (**c**) The BrdU results for evaluating cell proliferation with CEPO treatment after 8 h (*n* = 4). Statistical differences were determined by using One-way ANOVA and Two-way ANOVA with Bonferroni’s multiple comparison test. *p* value < 0.05 *, <0.001 ***, <0.0001 **** (**b**,**c**).

Increasing evidence indicates that Schwann cells facilitate the metabolism of regenerated axons, impacting myelination and the establishment of nodal domains, and serve as the principal glia in the peripheral nervous system [45,46,47,48]. Here, Schwann cell viability and proliferation experiments were conducted to investigate the potential of CEPO for peripheral nerve regeneration. As shown in Figure 3a,b, the live/dead experiment and CCK-8 assay validated that the high concentration CEPO (25 μg/mL) had superior biocompatibility for Schwann cells after 24 h, 48 h, and 72 h, especially at 3D, the relative cell viability in the 25 μg/mL CEPO group achieved an incredible 177% compared with the control group. Intriguingly, the low-concentration CEPO treatments (6.25 μg/mL and 12.5 μg/mL) did not have a significant effect on promoting the viability of Schwann cells. In contrast, low concentration of CEPO might exert an inhibitory effect on Schwann cell viability after 24 h. Furthermore, the proliferation of Schwann cells also plays a major role in nerve healing, as it guides the axonal growth and regeneration. Thus, the effect of CEPO on the proliferation of Schwann cells was also evaluated using the BrdU proliferation assay. Briefly, BrdU (bromodeoxyuridine or 5-bromo-2′-deoxyuridine) is an analog of the nucleoside thymidine. Upon cell proliferation or replication, BrdU is incorporated into the DNA instead of thymidine, and then detected using the anti-BrdU antibodies, following the manufacturer’s protocol. The absorbance value is proportional to the quantity of BrdU incorporated into cells. Our results showed that, among all concentrations, statistical significance was found in the group of 25 μg/mL (*p* < 0.01) compared to the control. Other groups of concentrations, including 6.25 μg/mL and 12.5 μg/mL, also displayed a higher average Schwann cell proliferation compared to the control, despite no statistical significance (Figure 3c).

#### 2.2.3. Schwann Cells Wound Healing and Migration Study with CEPO Treatments

**Figure 4:**
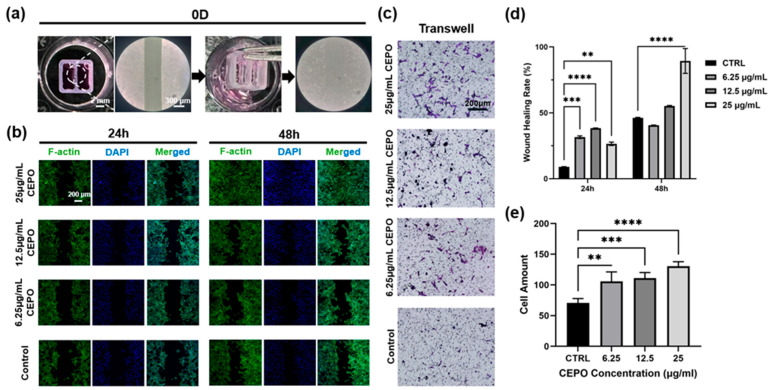
The wound healing and migration ability of Schwann cells with CEPO treatment. (**a**) The optical images of 0D samples seeded with Ibidi inserts. (**b**) Wound-healing results of the Schwann cells with different concentrations of CEPO at various time points (24 h and 48 h). (**c**) The Transwell migration ability of Schwann cells with CEPO administration was assessed using crystal violet staining at 24 h. (**d**) Quantitative analysis of the healing area/wounded area ratio in scratch healing tests at 24 h and 48 h (*n* = 4). (**e**) Quantitative analysis of the cell counts in Transwell migration assessments at 24 h (*n* = 4). Statistical differences were determined by using One-way ANOVA and Two-way ANOVA with Bonferroni’s multiple comparison test. *p* value < 0.01 **, <0.001 ***, <0.0001 **** (**d**,**e**).

Given that there are numerous inflammatory mediators or chemoattractants produced upon nerve injury, which facilitate the requirement for reparative cells. To study the impact of CEPO on the chemotaxis of Schwann cells, the wound-healing experiments with fluorescence staining and Transwell assays were conducted to assess the functional effects of CEPO on Schwann cells. Consistent with the cell viability experimental results, high-concentration CEPO had an obvious ability to enhance the wound healing of Schwann cells. As illustrated in Figure 4b, many more Schwann cells were observed at the margins and within the vacant area after 24 h in the CEPO-treatment groups compared to the control groups. Notably, the wound area was markedly reduced with significant healing in the 25 μg/mL group, while the 12.5 μg/mL CEPO treatment group also showed a substantial tendency for wound healing after 48 h. In addition, the Actin-Tracker Green staining results indicated that the cytoskeleton morphology of Schwann cells was well maintained after CEPO treatment. The ImageJ software (Version: 1.54g) was used to quantify the wound-healing rate according to the calculation of cell migration area, and the results demonstrated that the high-concentration CEPO treatment groups exhibited a significant difference in wound-healing rate compared to the control group after 48 h (Figure 4d).

In addition, the results of the Transwell migration study with ImageJ quantification of cell counts further confirmed a dose-dependent increase in the migration of the Schwann cells upon CEPO treatments, particularly at the 25 μg/mL concentration (*p* < 0.05), highlighting that CEPO positively enhances the migration of Schwann cells as a chemoattractant (Figure 4c,e). In summary, the aforementioned results indicated that CEPO, especially at 25 μg/mL, was conducive to cell growth, proliferation, and migration.

#### 2.2.4. In Vitro Immunofluorescence Staining of Schwann Cells upon CEPO Treatments

**Figure 5:**
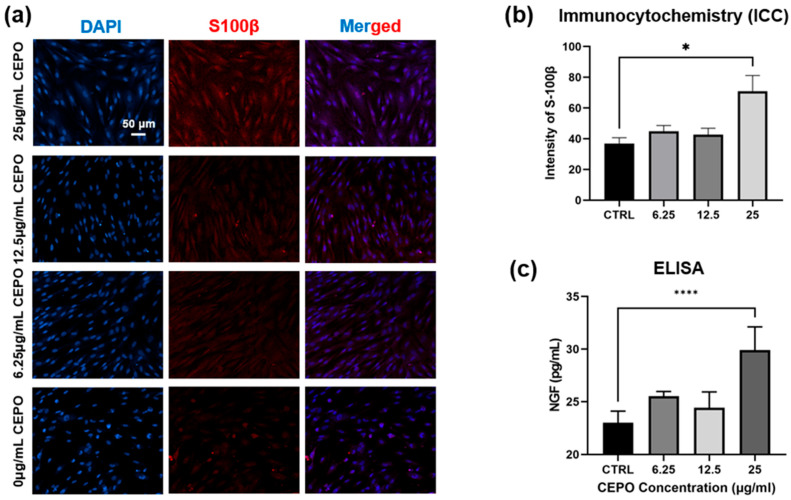
(**a**) The immunofluorescent staining images of the Schwann cells with different concentrations of CEPO administration after 24 h. (**b**) The intensity of S-100β fluorescence signals was quantitatively analyzed with ImageJ software (*n* = 4). (**c**) Quantitative graph depicting the Nerve growth factor (NGF) release from Schwann cells treated with different concentrations of CEPO (*n* = 4). Statistical differences were determined by using One-way ANOVA with Bonferroni’s multiple comparison test. *p* value < 0.05 *, <0.0001 **** (**b**,**c**).

The morphology of Schwann cells treated with CEPO is presented in Figure 5a. The immunofluorescent staining results demonstrated that Schwann cells grew well under CEPO administration after 24 h of culture, suggesting that CEPO provided a superior cultivating effect for nerve-related cell proliferation. Figure 5b indicated that the intensity of the S100β marker in the 25 μg/mL CEPO group was significantly higher than that of the group without CEPO treatment (*p* < 0.05). The stained images (Figure 5a) also illustrated that CEPO-administered Schwann cells exhibited greater spreading and an oriented morphology compared to those in the control group. Cells with CEPO treatments had a considerably greater length-to-width ratio and a lower orientation angle, indicating enhanced alignment growth. Furthermore, it was noted that, upon CEPO treatment, Schwann cells exhibited a greater number of neurites than those without CEPO, suggesting that CEPO played a beneficial role in enhancing possible neuronal repair from Schwann cells (Figure 5a).

#### 2.2.5. Nerve Growth Factors (NGF) Release from Schwann Cells

Nerve growth factor (NGF) release from Schwann cells promotes axonal regeneration. In this context, the amount of NGF release from the Schwann cells was evaluated using ELISA (Figure 5c). The results showed that NGF protein expression significantly (*p* < 0.05) increased in the 6.25 μg/mL and 25 μg/mL CEPO groups, with 25.53 and 29.91 pg/mL of NGF, respectively. Notably, the concentration of 25 μg/mL CEPO was found to have the most obvious influence on promoting neurotrophic protein secretion, where the levels of NGF protein expression increased approximately 1.3-fold compared with the control group. Considering all the results of the in vitro study, 25 μg/mL CEPO was chosen as the optimal concentration for further research.

### 2.3. In Vivo Animal Experimental Results with CEPO

#### 2.3.1. Functional Evaluations of Rats

On post-surgery day 21, Sprague Dawley rats were put on a digital walking gait to evaluate functional healing at a constant speed of 5 cm/s. Digigait recorded the rats’ walking exercise, including paw prints (toe spread, immediate toe spread, and print length), and the sciatic functional index (SFI) was analyzed as described above.

**Figure 6:**
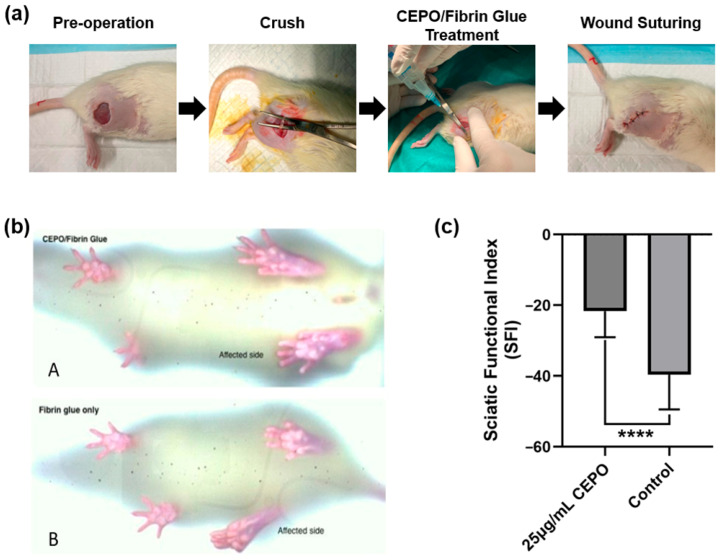
The functional assessment of regenerated nerves in the sciatic nerve defect rat model. (**a**) Surgery photographs depicting the application of CEPO-fibrin glue around the crushed sciatic nerve in the model rat. (**b**) Digigait camera screenshot demonstrating paw prints of rats. (**A**) Paw prints showing toe spread, print length, and intermediate toe spread of the affected (right) limb and unaffected (left) limb in group CEPO-fibrin glue; (**B**) Paw prints showing print length, toe spread, and intermediate toe spread of the affected (right) limb and unaffected (left) limb in group CEPO-fibrin glue. (**c**) Sciatic Functional Index comparing CEPO-fibrin glue group and Fibrin glue group on Day 21 (*n* = 4). A statistically significant difference was found between the two groups, with a *p*-value < 0.0001 (****).

As shown in Figure 6b, the Digigait images illustrated a comparable extent of toe spread and print length in the affected limb with the unaffected limb in the 25 μg/mL CEPO-fibrin glue group, in contrast to the fibrin glue group. Furthermore, as for the SFI calculation, the mean SFI was found to be −26.96 ± 11.90 in the group of 25 μg/mL CEPO-fibrin glue. Conversely, the average SFI was determined to be −36.12 ±7.60 in the control group treated only with fibrin glue. Student’s T-test showed a statistically significant difference between the two groups (*p* < 0.05) (Figure 6c). The difference in the SFI could be well demonstrated by the morphological change in the toe spread of the affected limb after nerve crush injury.

#### 2.3.2. In Vivo Immunocytochemistry (ICC)

To investigate whether localized delivery of CEPO could enhance neural healing, indicated by enhanced Schwann cell expression, which dominates Wallerian degeneration and serves as the leading edge of neural regrowth, sciatic nerves were harvested and incubated with an S100β primary antibody to assess the extent of healing.

**Figure 7:**
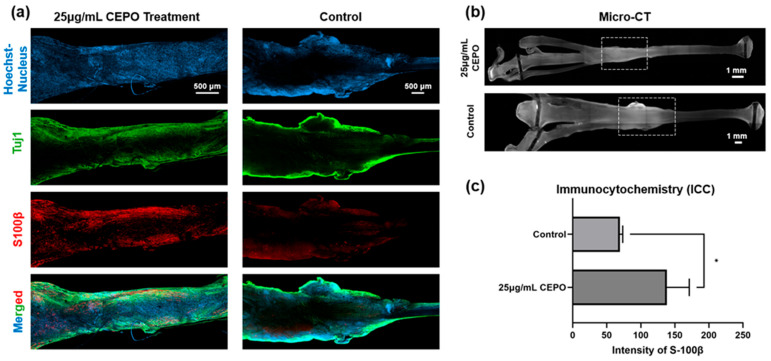
Improvement of axonal regeneration with CEPO treatment in the rat peripheral nerve injury models. (**a**) Fluorescence images of the nerve crush site in the CEPO-fibrin glue group and the fibrin glue group alone of whole-mounted staining samples. With S100β, Tuj-1, DAPI nuclear stain, and combined images. (**b**) The Micro-CT images of nerve crush site with CEPO-fibrin glue and fibrin glue alone (control) administrations. (**c**) Relative intensity of S100β comparing the CEPO-fibrin glue group and fibrin glue group on Day 21 (*n* = 3). A statistical difference was found between the two groups, *p*-value < 0.05 *.

Under this setting, both the 25 μg/mL CEPO-fibrin glue group and the fibrin glue (control) group were stained with S100β, Tuj-1, and DAPI, where the staining and micro-CT images are shown in Figure 7a,b. The crush site illustrated fewer stained nuclei, indicated by the Hoechst stain. At the crush site, there were strands of neurons growing across it, with band-like S100β-immunopositive signals, suggesting regenerating axons guided by the pioneer Schwann cells. The crush site in the control group did not show S100β-immunopositive signals, with scarce Tuj-1-immunopositive neuronal signals noted. In contrast, the relative intensity of S100β in the CEPO-fibrin glue group was found to be statistically significantly (*p* < 0.05) higher than the control group (Figure 7c). ICC statistical results showed that the intensity mean of S100β signals at the crush injury site reached 138.234 ± 32.91 in the CEPO-fibrin glue group, whilst the control group of fibrin glue only showed 69.184 ± 4.29.

## 3. Discussion

The key findings of the study were (1) A concentration of 25 μg/mL of CEPO enhances Schwann cell proliferation and migration in vitro. (2) A concentration of 25 μg/mL of CEPO enhances NGF expression from Schwann cells in vitro. (3) There was a statistically significant improvement in the sciatic functional index (SFI) of the rats that underwent CEPO treatment after nerve crush injury; and (4) there was statistically significantly higher expression in Schwann cells, with also axons discovered growing across the nerve crush injury site.

The role of Schwann cells in mediating peripheral nerve injury is undoubtedly essential. EPO is a medication used for chronic kidney disease and secondary anemic patients. It has been shown to cross the blood–brain barrier to reduce the size of the infarcts and is neuroprotective for ischemic injury of the CNS [49,50] and able to contribute to the recovery of motor function after ischemic stroke and spinal cord injury [51,52,53]. The signaling mechanism of EPO’s neuroprotective properties has been well understood [54,55]. However, the systemic side effects of EPO were also clearly identified. Aydin et al. showed that EPO treatment increased the risk of thrombotic events after administration of an intravenous bolus of EPO [56,57]. Multiple studies have also discussed minor adverse events induced by EPO administration, including headache, nausea, and general physical weakness. In this context, CEPO, a homolog of EPO, has been proven to show similar neuroprotective effects to EPO without exerting any hematopoietic activity. Leist et al. [35,36] suggested the neuroprotective effects of CEPO in various animal models of neurotoxicity, such as ischemic stroke, sciatic nerve compression, spinal cord compression, and peripheral diabetic neuropathy, while no erythropoietic effects were found after long-term usage of high doses of CEPO [35,37]. Nevertheless, research on the effects of CEPO on the peripheral nervous system remains limited, and there are few studies focusing on the local administration of CEPO in oral and maxillofacial surgery, aiming to promote facial peripheral nerve healing and functional regeneration.

This study, therefore, explored the impact of CEPO on the biological behavior of Schwann cells to evaluate the possibility of CEPO as a promising candidate for a pharmaceutical agent in the injured peripheral nervous system that could be locally applied in surgery. Upon nerve injury, Schwann cells demonstrate explicit plasticity induced by the injury signals, entailing reversal of myelin differentiation and phenotype differentiation from myelinated Schwann cells to reparative Schwann cells [58,59,60,61]. These biochemical events involving Schwann cells in response to injuries have been well studied and reported. The molecular events include down-regulation of myelin transcription factors Egr2 (Krox 20), Myelin Basic proteins (MBP), and myelin-associated glycoprotein (MAG) [62,63]. Moreover, Schwann cells differentiated into a novel repair phenotype by activation of transcription factor c-Jun [64,65] that upregulates neurotrophic proteins such as NGF that enhance axonal survival and growth, and the cytokines such as interleukin (ΙL)-1α, and tumor necrosis factor (TNF)-α [58,59]. More importantly, this phenotype of repair Schwann cells exhibits an elongated morphology, named the Bungner band, to act as a functional regenerative unit to provide guidance and chemotactic cues for the regenerating axons to grow from the proximal to the distal stump. The number of Schwann cells was found to be increased manifold at the injured distal stump to facilitate all these biochemical events. Therefore, proliferation and migration studies were targeted in our study to gain insight into how CEPO affects these crucial biological behaviors of Schwann cells. In our study, both proliferation and migration of Schwann cells were found to be enhanced under the impact of certain concentrations of CEPO. ELISA results also exhibited enhanced expression of the important neurotrophic factor of NGF under the influence of CEPO. All these in vitro results bring promising insights into how CEPO possibly facilitates peripheral nerve regeneration by enhancing Schwann cell biological activities, such as viability, proliferation, migration, and NGF secretion. Furthermore, from the in vitro part of our study, it was found that CEPO significantly enhances Schwann cell growth and migration, suggesting its potential for nerve repair. We also noticed that CEPO-treated Schwann cells tend to grow in a direction, as seen in Figure 3b and Figure 4a. The directional growth in our CEPO-treated Schwann cells may result from biochemical cues mediated by CEPO, which enhance the development of a polarized morphology, a fundamental feature of adequate axonal regeneration. This polarization can help Schwann cells migrate toward injury sites and improve their support for peripheral nerve repair. Our in vivo studies using rat models with sciatic nerve crush injuries reinforce this information; the CEPO group shows better healing and more organized nerve regeneration than controls. These results highlight CEPO’s ability to augment cell proliferation and guide Schwann cell behavior, both of which are critical for successful nerve repair. An extended analysis of the mechanisms underlying this directional growth will be essential to fully understand the consequences of CEPO treatment in regenerative medicine.

In addition, from our in vivo study, the SFI of the CEPO-treated rats was found to have improved significantly when compared to the control group. SFI is the preferred method to assess the effectiveness of CEPO treatment in this study. Experiments on peripheral nerve regeneration are almost exclusively performed using walking track analysis models. The SFI index is regarded as reliable and accurate for assessing functional changes following injury and the subsequent return of sensory and motor functions, and allows fair comparisons with results from other studies. This study showed that CEPO remarkably improved the physiological healing of the rats after sciatic nerve crush over a 21-day review period compared to the control group without CEPO. Furthermore, S100β and Tuj-1 were chosen to be the primary antibodies to quantify Schwann cells and neuronal structures. S100β immune-positive signals in the experimental group of CEPO-fibrin glue were found to be significantly higher than those in the control group. There were Tuj-1 immunopositive signals indicating regrowing axons across the injury site, with band-like Schwann cells immunopositive signals guiding proximal to the regrowing axons. These findings suggest that CEPO delivered locally via fibrin glue may enhance Schwann cell biological activities and associated axonal regrowth upon injury in vivo, which may further provide chemotactic cues for peripheral nerve repair. This in vivo study also showed that CEPO, through localized delivery with fibrin glue applied at the nerve crush injury site, could promote physiological healing in rats, with significant improvement in sciatic functional index after 21 days. The difference could possibly be explained histologically by the enhancement of Schwann cell biological activities that guide the axons to regenerate across the injury site. The current study sets the foundation for a larger in vivo study to determine the neuroprotective effect of locally applied CEPO of various dosages in nerve injury models.

Previous work has examined how EPO and its non-erythropoietic analog CEPO behave in different models of neural and tissue injury. Leist et al. originally demonstrated that CEPO retains the tissue-protective properties of EPO across multiple animal models—including ischaemic stroke and sciatic nerve compression—while producing no measurable erythropoietic response even at high doses. In hippocampal slice cultures subjected to oxygen-glucose deprivation and NMDA-induced excitotoxicity, CEPO provided neuroprotection at least comparable to that of EPO [66]. Similar findings were reported in a spinal cord injury model, where both EPO and CEPO improved neurological outcomes to a similar degree, further supporting the notion that the neuroprotective and erythropoietic properties of EPO can be successfully dissociated [67]. Beyond the nervous system, CEPO has also been shown to outperform EPO in experimental acute kidney injury, offering organ protection with a reduced hematological side-effect profile [68]. Taken together, these findings suggest that CEPO represents a tissue-protective derivative of EPO with a more favorable safety profile. However, although there have been numerous studies exploring the influence of CEPO on the nervous system, most research concentrates on CNS diseases, such as Alzheimer’s disease and spinal cord injury, rather than peripheral nerve repair. This study provides the potential for exploring the possibility of developing CEPO as a therapeutic option for patients who are at high risk of developing neurosensory deficits from surgery. Crush injury, rather than sectioning, was administered in this experiment to imitate neurapraxia without any disruption in endoneurium, perineurium, and epineurium, to imitate the common injury mode commonly encountered in the oral and maxillofacial fields, such as third molar extractions and orthognathic surgery. Emerging evidence regarding the neuroprotective effects of CEPO holds significant promise for surgical practice, especially within the fields of oral and maxillofacial surgery and orthognathic procedures. These surgeries often require intricate manipulation of facial structures and vital nerves, thereby presenting a considerable risk of nerve injury. Damage to the inferior alveolar or mental nerves can result in sensory deficits, chronic pain, and extended recovery periods, all of which may profoundly impact a patient’s quality of life. In our study, local administration of CEPO enhanced Schwann cell viability, migration, and NGF secretion in vitro, and improved functional and histological parameters after sciatic nerve crush, which is in keeping with the established neuroprotective activity of CEPO and further extends its application to the context of peripheral nerve repair delivered via a localized approach. The rigorous in vitro screening identified 25 μg/mL as the optimal therapeutic dose of CEPO for Schwann cell activation. Establishing this specific dose provides a critical, previously absent foundation for the future development of sustained-release, local drug delivery systems tailored for post-surgical peripheral nerve repair.

Although the results of this study reveal that CEPO has certain potential and prospects in promoting the recovery and regeneration of peripheral nerves, it is expected to become one of the effective drugs for treating nerve injuries caused by surgery, several limitations must still be acknowledged. Firstly, CEPO was identified only by SDS-PAGE electrophoresis, whereas adding more detailed characterization, like LC-MS or MALDI-TOF, might be more convincing in the study. In addition, while the chemical carbamylation process is well-documented to eliminate the erythropoietic activity of EPO, future studies should include direct hematological evaluations to empirically confirm the absence of erythropoietic effects in the specific CEPO preparation. Moreover, the in vivo experiments were conducted with a relatively small sample size and a short follow-up period, which restricts the ability to assess the long-term durability of functional recovery. There is currently a lack of comprehensive toxicity and safety evaluations, which are critical prerequisites before CEPO can be considered for clinical application as a post-surgical treatment. Furthermore, the absence of mechanistic validation on signal pathways limits the depth of this research. It has been reported that the peripheral nerve regeneration is related to PI3K/Akt [69], MAPK/ERK [70] and JNK/c-Jun [71] signaling cascades. Because this study did not investigate how CEPO regulates or activates these specific pathways, its exact molecular targets remain unclear. Future work would specifically test whether CEPO modulates these signaling cascades in Schwann cells in vitro and in a relevant nerve injury model in vivo. Addressing these limitations through larger, long-term in vivo studies, rigorous toxicological assessments, and targeted molecular investigations will be essential steps before translating these findings into clinical practice.

CEPO’s ability to enhance Schwann cell proliferation, migration, and nerve growth factor (NGF) expression underscores its potential as an adjunctive therapy for postoperative recovery. By upregulating NGF—a molecule essential for neuronal repair—CEPO may mitigate nerve-related complications, thereby facilitating more rapid and effective healing. The prospect of developing CEPO as a therapeutic agent is highly encouraging. However, the traditional pharmacological treatments, such as administration of CEPO alone, might exhibit some limitations, including inadequate targeting, rapid drug elimination from the circulatory system, and limited therapeutic efficacy, potentially causing some side effects in clinical applications [72,73]. Therefore, implementing a local drug delivery system with sustained-release capabilities of CEPO may provide prolonged neuroprotective effects, supporting continuous nerve regeneration, and could be considered as a prospective research direction based on this study. Such an approach could enhance reparative Schwann cell biomarkers and foster a regenerative microenvironment, thereby maximizing nerve recovery. A sustained-release mechanism has the potential to transform postoperative care by delivering ongoing support for nerve repair precisely at the surgical site in the future.

## 4. Materials and Methods

### 4.1. Chemical Synthesis of CEPO and Characterization

CEPO was prepared as described previously [35,36,37]. Recombinant EPO (rhEPO) was purchased from Peprotech (ThermoFisher, Waltham, MA, USA). Briefly, 50 μg of rhEPO was dissolved in 1 mL of deionized water and mixed with 1 mol/L sodium borate (pH 8.8). The solution was further reacted with 1 mol/L potassium cyanate (KOCN, ThermoFisher, Waltham, MA, USA), and then incubated in a 37 °C water bath overnight. To eliminate the impact of the degree of carbamylation on the study, an excess of cyanate was used in the CEPO preparation process [74]. The residual KOCN was removed by dialysis using dialysis cassettes (Slide-A-Lyzer, 10 kDa, ThermoFisher, Waltham, MA, USA). The reaction mixture was loaded into the dialysis cassettes and dialyzed under stirring at 4 °C against Milli-Q water and 1 L of the salt solution (20 mmol/L sodium citrate and 0.1 mol/L NaCl, pH 6.0) overnight, respectively. The remaining solution in the dialysis cassettes was subsequently retrieved. To facilitate storage and future use, the powder form of the content was obtained using the freeze-dry method and stored at −20 °C.

Characterization of the complete chemical synthesis of CEPO from EPO was identified by electrophoresis. Despite advanced techniques such as LC-MS or MALDI-TOF providing higher-resolution structural characterization, the primary objective in this study was to confirm the successful carbamylation of EPO at the level of lysine modification for routine batch-to-batch verification. Therefore, the established functional assay originally described by Leist et al. was adopted to do CEPO characterization [36]. EPO is composed of 165 amino acids and contains free amino groups (-NH2) from 8 lysine residues available for carbamylation by reacting with cyanate. Since endoproteinase Lys-C specifically cleaves at unmodified lysine residues but cannot cleave fully carbamylated lysines, resistance to this enzyme serves as a reliable indicator of successful conversion. To perform this assay, the freeze-dried powder of rhEPO and CEPO was dissolved in deionized water and incubated with endoproteinase Lys-C (sequencing grade, Sigma-Aldrich, St. Louis, MO, USA) overnight at 37 °C. The molecular weight of native rhEPO is approximately 34 kDa, and upon enzymatic cleavage, it yields chains of approximately 16 kDa. Consequently, successful conversion into CEPO protein is confirmed by the retention of a single band at approximately 31–34 kDa following Lys-C incubation, whereas unreacted rhEPO is expected to show a cleaved fragment at approximately 16 kDa. The digested solution was then electrophoresed using 12% sodium dodecyl sulfate-polyacrylamide gel electrophoresis (SDS-PAGE) to confirm the final result of the carbamylated modification.

### 4.2. In Vitro Experiments

To ensure rigorous methodology and minimize bias, all in vitro data acquisition (including microscopy imaging and microplate readings) and subsequent quantitative analyses were performed by investigators who were blinded to the experimental group assignments.

#### 4.2.1. Culture and Characterization of Schwann Cells

Rat Schwann cell lines (SCs, ECACC, SCL 4.1/F7) were used in the experiments. SCs from liquid nitrogen storage were thawed and transferred to a 10 mL centrifuge tube (BD Biosciences, San Jose, CA, USA). The freezing medium was diluted with 5 mL of SC culture medium, comprising MEM-Alpha (ThermoFisher, Waltham, MA, USA) supplemented with 5% Fetal Bovine Serum (FBS, Biosera, Ringmer, UK), 100 ng/mL of beta-Heregulin (βH, Sigma-Aldrich, St. Louis, MO, USA), 20 ng/mL of basic fibroblast growth factor (bFGF, PeproTech^®^, ThermoFisher, Waltham, MA, USA), 5 ng/mL of platelet-derived growth factor-AA (PDGF-AA, Sigma-Aldrich, St. Louis, MO, USA) and 5 μΜ of forskolin (Sigma-Aldrich, St. Louis, MO, USA) to foster differentiation of mature SCs to minimize the toxicity of DMSO. The cells were then centrifuged (10,000 rpm, 5 min, 25 °C). The supernatant was then removed, and the cell pellets were resuspended in 2 mL of culture medium. They were plated on the culture dish with a density of 25,000–30,000 cells/cm^3^. Upon reaching confluence of about 80%, the presumptive SC colonies were detached by TrypLE Express Enzyme (ThermoFisher, Waltham, MA, USA), incubated at 37 °C for 3 min, and were passaged. After 3 min, the trypsinated solution was neutralized with culture medium. SCs are recovered by centrifugation (10,000 rpm, 5 min, 25 °C). This passage was counted as an arbitrary passage 1, and SCs within passages 4–6 were used for later experiments. Furthermore, the Schwann cells were further characterized and confirmed by immunocytochemistry (Figure S1).

#### 4.2.2. Cell Viability and Biocompatibility Assessments

The cell counting kit-8 (CC-K8) assays were employed to assess the Schwann cell viability after different concentrations of CEPO treatments. Briefly, the Schwann cell suspension was seeded into the 96-well cell culture plates (Biofil, Jet Bio-Filtration Co., Ltd., Guangzhou, China) at a density of 3 × 10^4^ cells/mL, and each well received 100 μL cell suspension, followed by placing the well plates into the cell culture incubator for 6 h. After the Schwann cells adhered to the bottom of the plates completely, the original medium was removed, and the new cell medium containing different concentrations of CEPO (standard Schwann Cell medium without CEPO, serving as the untreated vehicle control) was added to each well. The viability of Schwann cells was assessed after 24, 48, and 72 h using the Enhanced CCK-8 Assay kit (Beyotime, Shanghai, China). Absorbance at 450 nm was measured using a multi-labeled microplate detector. Additionally, Live (green)/dead (red) tests were conducted on the same density of Schwann cells at the same time points by using the Calcein/PI Cell Viability and Cytotoxicity Detection Kit (Beyotime, Shanghai, China). Specifically, Schwann cells cultivated for 1D, 2D, and 3D were rinsed twice with PBS, treated with the Calcein/PI staining solutions, and incubated at 37 °C, 5% CO_2_ for 30 min in the absence of light. Both the live and dead cells were examined using an inverted fluorescent microscope (Eclipse Ti-S, Nikon Instruments, Tokyo, Japan).

#### 4.2.3. In Vitro Proliferation Test

The 5′-Bromo-2′-deoxyuridine (BrdU) Cell Proliferation Assay Kit (Cell Signaling Technology, Danvers, MA, USA) was used to assess proliferating Schwann cells by detecting BrdU incorporation into cellular DNA throughout proliferation with an anti-BrdU antibody. In brief, SCs divided into treatment groups receiving different concentrations of CEPO (25, 12.5, 6.25, and 0 µg/mL) were seeded onto 96-well plates. Cell seeding density was 5 × 10^3^ cells/well. 1× BrdU labeling medium was added to the culture medium, and SCs were cultured for 24 h. After removing the labeling medium, cells were fixed, and the DNA was denatured with 100 µL/well of fixing/denaturing solution for 30 min. 100 µL/well of BrdU mouse mAb was then added to detect the incorporated BrdU, and incubated for 1 h. The solution was removed, and the plate was washed 3 times with 1× WASH Buffer. After that, 100 µL/well of Anti-mouse IgG, HRP-conjugated secondary antibody was then used to identify the bound detection antibody at room temperature for 30 min, followed by further washing with the WASh Buffer 3 times. The HRP substrate TMB was added and incubated for 30 min to develop color. 100 µL of STOP solution was added per well. Colorimetric readings were taken with absorbance at 450 nm and analyzed by Multimode Microplate Reader (SpectraMax ID5, Molecular Devices, San Jose, CA, USA).

#### 4.2.4. In Vitro Wound Healing and Migration Evaluations

As for the wound-healing evaluation, the transverse scratches were created to simulate artificial wounds among Schwann cells using ibidi Culture-Inserts (ibidi GmbH, Gräfelfing, Germany). In brief, 100 µL of cell suspension containing 8000 Schwann cells was seeded on each side of the ibidi Culture-Inserts container in the 24-well cell plates (Biofil, Jet Bio-Filtration Co., Ltd., Guangzhou, China). The ibidi inserts were removed after overnight incubation, and the fresh SCs medium, which contained different concentrations of CEPO (25 µg/mL, 12.5 µg/mL, and 6.25 µg/mL), was applied to the Schwann cells, respectively (Figure 4a and Figure S2). Subsequently, 1× PBS was used to gently wash the samples three times to remove the suspended cells, followed by staining with Actin-Tracker Green 488 (Beyotime, Shanghai, China) and DAPI (Sigma-Aldrich, St. Louis, MO, USA) after 24 and 48 h of culture. Eventually, cell morphology was observed, and micrographs were acquired by the confocal microscope (ZEISS LSM 980, Zeiss, Jena, Germany). The wound-healing rate was obtained by quantification of the healing area using ImageJ V1.54g (National Institutes of Health, Bethesda, MD, USA).

Moreover, transwell experiments with crystal violet staining were performed to further assess the promoting effect of CEPO on the migration ability of Schwann cells. Briefly, Schwann cells were seeded on top of the chamber and incubated on a Transwell cell insert (8 µm, Beyotime, Shanghai, China) with a density of 1 × 10^4^ cells/cm^2^ for 24 h. The bottom chamber of the transwell contained Schwann cell media along with varying concentrations of CEPO. Experimental groups involved the use of SC medium as described in the previous section, containing CEPO of varying concentrations of 25 µg/mL, 12.5 µg/mL, and 6.25 µg/mL, while the control group received no CEPO (standard Schwann Cells medium serving as the untreated vehicle control). During the incubation time, Schwann Cells were expected to migrate through the pores along the gradient to the bottom side of the transwell insert membrane. The non-migrated cells on the upper side of the membrane were carefully removed by wiping with a cotton bud after 48 h. Medium was removed, and the cells adhered onto the insert membrane were fixed with PFA in 1× PBS for 20 min, followed by rinsing with 1× PBS for three times, 5 min each. The membrane was stained using crystal violet (Beyotime, Shanghai, China) for 10 min. Digital images were taken using an Eclipse LV100 polarizing microscope (Nikon Instruments, Tokyo, Japan) and analyzed using ImageJ V1.54g (National Institutes of Health, Bethesda, MD, USA).

#### 4.2.5. In Vitro Immunofluorescence Staining and Determination of Nerve Growth Factor Release

Schwann cells with the density of 1 × 10^5^ cells/mL were seeded into 24-well plates and cultured under standard conditions (37 °C, 5% CO_2_) until they reached approximately 60–70% confluence. Cells were then exposed to CEPO at different concentrations of 25 µg/mL, 12.5 µg/mL, 6.25 µg/mL, and 0 µg/mL (as the untreated vehicle control) for 24 h. Following treatment, Schwann cells were fixed in 4% Paraformaldehyde (PFA) in 1× PBS for 20 min, followed by rinsing with 1× PBS three times, 5 min each. After that, a blocking buffer was used to block non-specific binding sites, which was composed of 1× PBS with 3% (*v*/*v*) normal goat serum (NGS, ThermoFisher, Waltham, MA, USA) and 0.3% (*v*/*v*) Triton™ X-100 (Sigma-Aldrich, St. Louis, MO, USA), for 30 min. Schwann cells were detected by the primary antibody of polyclonal rabbit anti-human S100β (1:200, Invitrogen, ThermoFisher, Waltham, MA, USA). Primary antibodies were incubated at 4 °C overnight. After being washed with 1× PBS three times for 5 min each, cultures were incubated with secondary antibody of Alexa 594-conjugated goat anti-rabbit (1:250, Invitrogen, ThermoFisher, Waltham, MA, USA). Hoechst Stain (DAPI, 10 μg/mL, Sigma-Aldrich, St. Louis, MO, USA) was added to stain nuclei. Culture dishes were captured as digital images of the fluorescence emission at 594 nm, and digital images were analyzed. The intensity of the S100β marker in different groups was quantified by ImageJ Software (V1.54g, National Institutes of Health, Bethesda, MD, USA).

Rat NGF beta Enzyme-linked immunosorbent Assay (ELISA) Kit (Invitrogen, ThermoFisher, Waltham, MA, USA) was used to analyze the level of nerve growth factors (NGF) in Schwann cell culture under treatment of CEPO for 48 h. In brief, after treating with trypsin and washing with 1× PBS, Schwann cells were suspended in SC medium as described in the previous section, and different concentrations of CEPO (25 µg/mL, 12.5 µg/mL, 6.25 µg/mL, and 0 µg/mL) were added. The cells were seeded in a 6-well plate and incubated for 48 h under the conditions of 37 °C and 5% CO_2_. The cell culture supernatant was collected in each cell well after 48 h. Subsequently, 96-well ELISA plates were incubated with the samples and standards for 2 h at room temperature after being coated with the diluted Capture Antibody overnight and blocked. 100 μL of the working Streptavidin-HRP dilution was then added to each well, followed by covering the plate and incubating for 20 min. Afterward, the Substrate Solution was added to each well, with another 20 minutes of incubation. Eventually, the absorbance at 450 nm was determined using the Multimode Microplate Reader (SpectraMax ID5, Molecular Devices, San Jose, CA, USA) after the addition of Stop Solution to the wells.

### 4.3. In Vivo Animal Experiments

#### 4.3.1. Construction of In Vivo Sciatic Nerve Defect Animal Model

Adult Sprague-Dawley (SD) rats of 10–18 weeks (*n* = 8, 280–320 g) were used in the experiment. They were bred and provided by the Center for Comparative Medicine Research (CCMR) of the University of Hong Kong (HKU). The animal study was approved by the Committee on the Use of Live Animals in Teaching and Research (CULATR) of the HKU, numbered 5804-21, and all procedures were conducted in strict accordance with institutional and ethical guidelines for animal care and use. The sample size (*n* = 4 per group) was justified based on a preliminary power analysis (α = 0.05, power = 80%) derived from expected effect sizes observed in previous similar nerve regeneration models, ensuring statistical validity while strictly adhering to the 3Rs (Replacement, Reduction, Refinement) principles to minimize the number of animals used.

Briefly, all rats were anesthetized using 75 mg/kg ketamine and 10 mg/kg xylazine. The intensity of anesthesia was monitored by foot pinch. The surgical area on the dorsal side of the right hind limb was shaved with an electric shaver, and the skin was disinfected locally with 80% ethanol and betadine alternatingly. The great trochanter was palpated, and the femur was located. The sciatic nerve defect was introduced by first making an incision 1 cm distal to and 0.5 cm caudal to the femur (trochanter major) to expose biceps femoris, then separating the superficial muscle layer with scissors and blunt dissection until the sciatic nerve was visible. The sciatic nerve was separated from the fascia carefully and lifted with curved end forceps. The sciatic nerve segment in the central area underneath the gluteus maximus, 1 cm before the trifurcation, was crushed with Spencer Wells artery forceps at the 3rd click, twice, for 20 s each time. Evident limb fibrillation in the right thigh and paw confirmed that the sciatic nerve was crushed. The crushed site was then treated with CEPO-fibrin glue (Tisseel, Baxter, Rome, Italy) or fibrin glue alone, accordingly. Single interrupted sutures were placed to close the muscle layer and skin using round-bodied Vicryl (4-0) and reverse-cutting Ethilon (5-0), respectively.

All rats tolerated the surgery well and were returned to individual caging. Animals were placed in a clean and dry area for clear observation post-surgically. 0.05 mg/kg Buprenorphine and 1 mg/kg Meloxicam were used as analgesics consecutively for 3 days post-surgically. Wound dehiscence at surgical sites was carefully monitored to ensure that all rats were free of abnormal behavior signs.

#### 4.3.2. In Vivo Experimental Design

Rats were randomized into 2 groups based on the types of materials locally injected at the nerve crush site. Randomization was performed using a computer-generated random number sequence to prevent selection bias. Eight rats were randomly assigned to receive a single injection of CEPO with fibrin glue (group CEPO-fibrin glue, *n* = 4) or fibrin glue alone (group fibrin glue, *n* = 4). To ensure rigorous methodology, the study was conducted in a double-blinded manner; the surgeons performing the procedures, the personnel providing postoperative care, and the investigators conducting the physiological assessments and data analyses were all blinded to the treatment group assignments. For both groups, rats were assessed physiologically and then sacrificed on Day 21, respectively. According to the in vitro experimental results, 25 μg/mL of CEPO was applied locally at the nerve crush site (Figure 6a).

#### 4.3.3. Functional and Physiological Evaluations

On Day 21 after surgery, the physiological function of the affected hindlimb of rats in different experimental groups was evaluated by a walking track test. Rats were put onto the Digigait (MouseSpecifics Inc., Framingham, MA, USA) for walking exercise at a constant speed of 5 cm/s with no inclination.

Paw prints of both left and right hind limbs were recorded by the Digigait in-built camera and analyzed by the Digigait software V12.2. Footprints that are indistinguishable, with fewer than five toe marks, or overlapped with other footprints were excluded. The average of footprint parameters of healthy and wounded feet was obtained, and the sciatic functional index (*SFI*) was calculated by the following Walking Track Test formula [75]:

$$SFI=-38.3\left(\frac{EPL-NPL}{NPL}\right)+109.5\;\left(\frac{ETS-NTS}{NTS}\right)+13.3\;(\frac{EITS-NITS}{NITS})$$

where Print length (*PL*), Toe Spread (*TS*), and intermediate toe spread (*ITS*) of the experimental (*E*) and normal side (*N*) were measured respectively and incorporated into Bain’s formula (1989).

#### 4.3.4. Histological and Immunocytochemical Analysis

After the walking track test, sciatic nerves in the affected hind limb of different groups were harvested (Figure S3), and the epineurium was removed to allow better penetration of the antibody. Fixation was done with 4% paraformaldehyde (PFA) for 24 h at 4 °C, followed by rinsing with 1× PBS three times for 10 min each. Non-specific binding sites on the sample slides were blocked with blocking buffer, comprising PBS with 3% (*v*/*v*) normal goat serum (NGS, ThermoFisher, Waltham, MA, USA) and 0.3% (*v*/*v*) Triton^TM^-X100 (Sigma-Aldrich, St. Louis, MO, USA) at 4 °C overnight. Primary recombinant rabbit anti-S100β antibody (1:150, Abcam, Cambridge, UK) and mouse anti-tubulin beta 3 (Tuj-1) antibody (1:200, Sigma-Aldrich, St. Louis, MO, USA) were used to detect Schwann cells and neurons, respectively. Both primary antibodies were incubated at 4 °C for 72 h. After incubation, the samples were washed with PBS three times for 15 min each and then washed in PBS overnight with a change in PBS every 1 h. Corresponding secondary antibody for Tuj-1 was Alexa-488-conjugated goat anti-mouse (1:500, ThermoFisher, Waltham, MA, USA), and for S100β was Alexa-594-conjugated goat anti-mouse (1:250, ThermoFisher, Waltham, MA, USA). Two to three drops of Anti-fading medium containing DAPI were placed on the tissue samples and incubated at 4 °C. Samples were then washed in PBS, and nerves were mounted in Citifluor Antifadent Mountant Solutions (Agar Scientific, Stansted, UK) for confocal imaging and captured as digital images of the fluorescence emission at 488 nm and 594 nm.

### 4.4. Statistical Analysis

For the experiments containing two groups, Student’s T-tests were used to compare the means of experimental results. One-way ANOVA and Two-way ANOVA with Dunnett’s multiple comparison analysis were applied for experiments containing more than 2 groups. A *p*-value < 0.05 was considered significantly different. The statistical analysis and graphs were performed using GraphPad Prism V10.1 (GraphPad Software Inc, San Diego, CA, USA). All the in vitro experiments were performed in triplicate at three independent time intervals.

## 5. Conclusions

In summary, the present study demonstrates the potential of CEPO in directing and repairing peripheral nerves at present study. Our in vitro investigations confirmed that CEPO, especially high-concentration CEPO, has the capability to enhance the orientation growth, proliferation, and migration of Schwann cells, while promoting the expression of Nerve Growth Factors (NGF) simultaneously. The in vivo experiments further revealed that CEPO has a positive impact on significant functional improvement and peripheral nerve repair. However, while these phenotypic and functional outcomes are encouraging, the specific intracellular signaling mechanisms through which CEPO exerts these effects remain to be fully elucidated. Future studies investigating these underlying molecular pathways will be essential to comprehensively evaluate and validate CEPO as a viable therapeutic approach for improving nerve regeneration and restoring locomotor function after peripheral nerve injury.

## Data Availability

Data are available from the corresponding author on reasonable request.

## Supplementary Materials

The following supporting information can be downloaded at: https://www.mdpi.com/article/10.3390/ijms27104434/s1.
