# Supplementary material for: Insights into the Effects of Carbamylated Erythropoietin on Schwann Cells in Peripheral Nerve Injury
Source: Int J Mol Sci. 2026 May 15;27(10):4434. doi: 10.3390/ijms27104434 (PMC13208020; doi:10.3390/ijms27104434)
Supplement: Supplementary file 1 [file ijms-27-04434-s001.zip › ijms-4244583-supplementary.pdf]

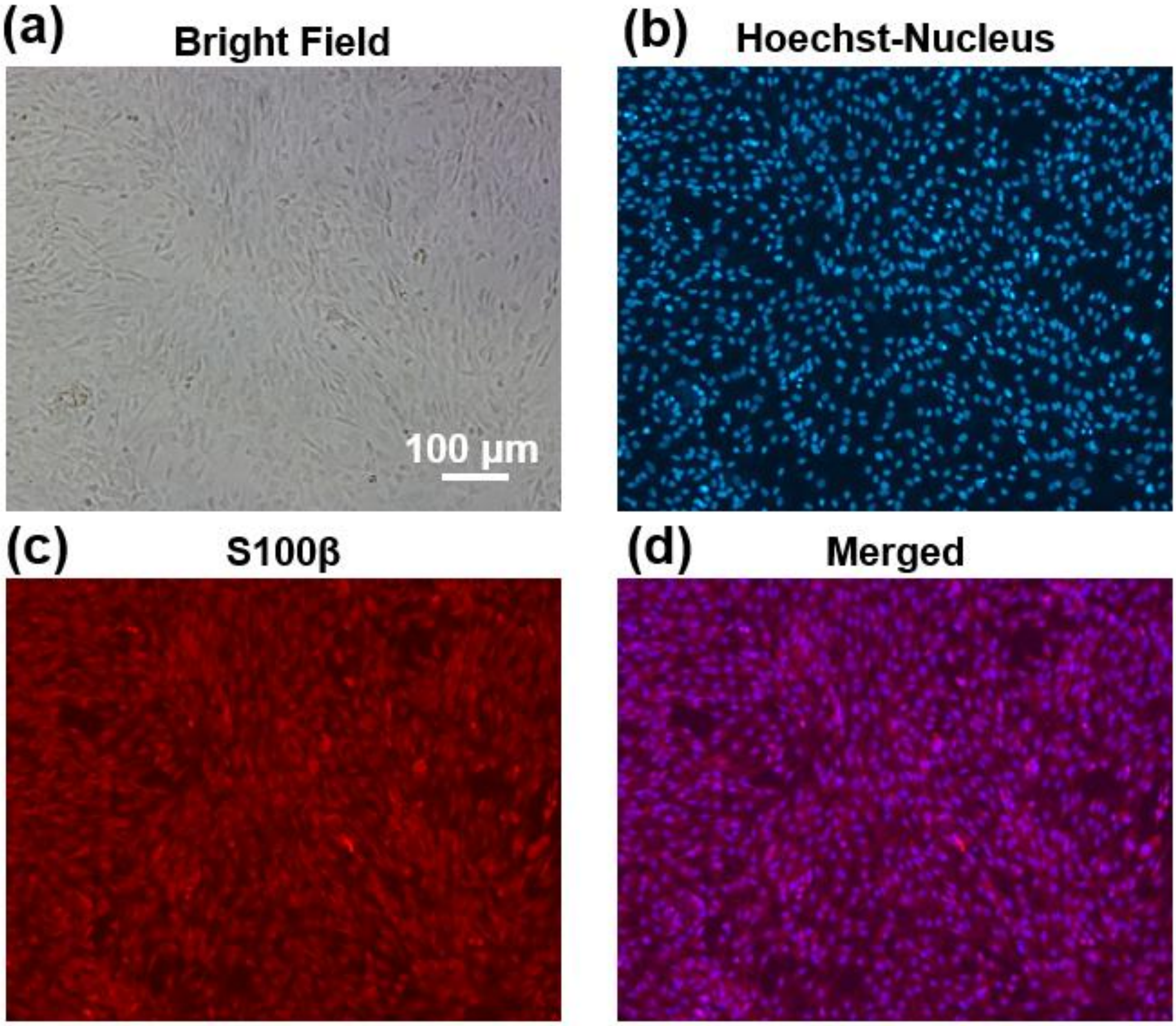

Figure S1. Confirmation of Schwann cells.

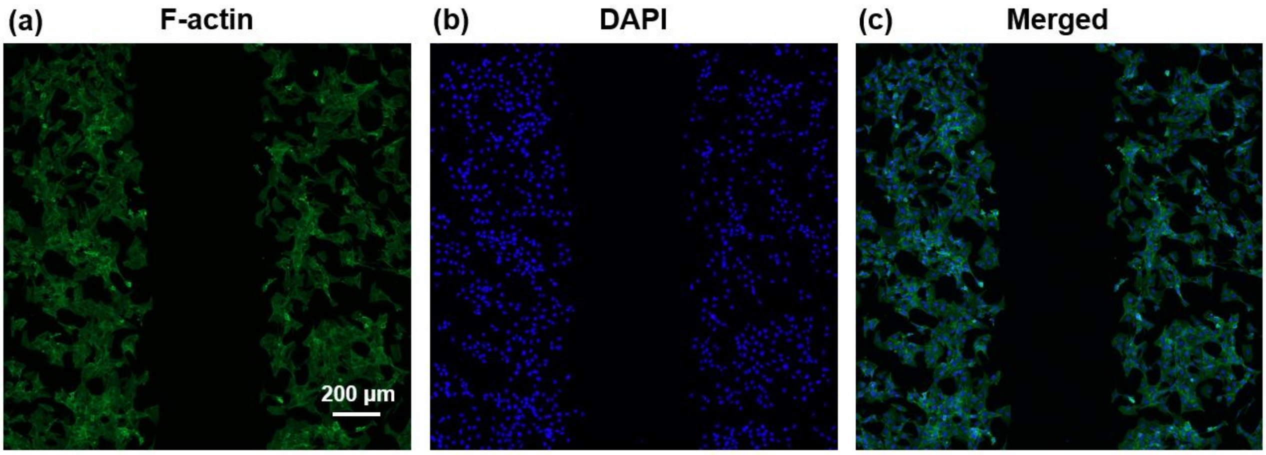

Figure S2. Immunofluorescence staining of 0D sample in the wound healing test.

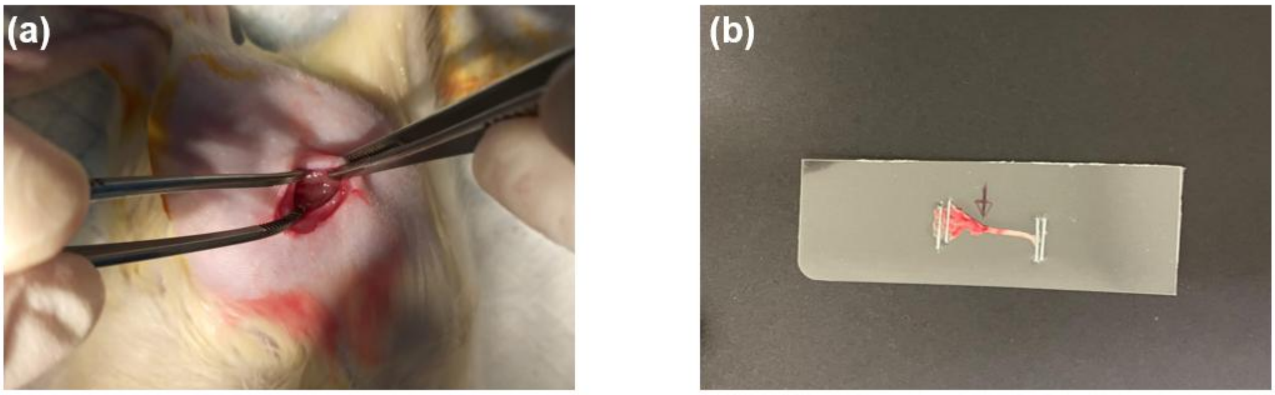

Figure S3. The surgery and sciatic nerve extraction images.
